# An innovative framework based on GMV-ALFF multi-modal MRI and Gaussian data augmentation for psychiatric disorder classification

**DOI:** 10.3389/fncom.2026.1835559

**Published:** 2026-09-30

**Authors:** Wenjing Zhu, Xiaoying Jiang

**Affiliations:** Affiliated Mental Health Center & Hangzhou Seventh People's Hospital, Zhejiang University School of Medicine, Hangzhou, China

**Keywords:** ALFF, bipolar disorder, convolutional neural network, D-S evidence theory, GMV, MRI, multi-modal fusion, schizophrenia

## Abstract

**Objective:**

Schizophrenia (SZ) and bipolar disorder (BD) exhibit significant overlap in clinical symptoms, posing substantial challenges to accurate diagnosis. This study proposes an innovative multi-modal fusion framework based on convolutional neural networks (CNNs), integrating structural and functional MRI biomarkers-gray matter volume (GMV) and amplitude of low-frequency fluctuation (ALFF).

**Methods:**

Total 31 patients with schizophrenia, 48 patients with bipolar disorder, and 38 healthy controls (HC) were enrolled and underwent resting-state fMRI scans. The framework comprises three core components: Gaussian data augmentation to mitigate the limitation of small sample sizes; a dedicated CNN architecture for processing GMV and ALFF features to generate class probability outputs for SZ, BD, and HC; and DempsterShafer (D-S) evidence theory for robust multi-modal decision fusion.

**Results:**

Extensive 5-fold cross-validation confirms the framework's efficacy: it achieves superior performance metrics (Accuracy: 0.9656 ± 0.0324, Precision: 0.9698 ± 0.0319, Recall: 0.9656 ± 0.0324, F1-Score: 0.9661 ± 0.0324) and outperforms traditional machine learning methods (SVM, Random Forest, XGBoost, KNN).

**Conclusion:**

The findings demonstrate that the proposed multi-modal fusion approach enhances the ability to differentiate among SZ, BD, and HC compared to unimodal classification methods, providing a reliable technical support for the auxiliary diagnosis of psychiatric disorders.

## 1 Introduction

Schizophrenia (SZ) and bipolar disorder (BD) are severe psychiatric disorders characterized by significant symptomatic overlap, which poses considerable challenges to clinical differentiation (Eken et al., 2022). While SZ is typically manifested by hallucinations, delusions, and cognitive disorganization, BD is defined by alternating manic and depressive episodes (Cano-Ruiz et al., 2020). Notably, both disorders share core clinical features, such as mood instability and cognitive impairment, frequently resulting in diagnostic uncertainty–especially during acute disease phases. This diagnostic dilemma carries substantial clinical implications, as misclassification can significantly compromise treatment outcomes given the distinct therapeutic strategies required for SZ and BD. To address this unmet clinical need, interdisciplinary approaches have been sought to develop more objective and accurate diagnostic tools. Among these approaches, computational psychiatry has emerged as a promising solution to address these diagnostic challenges by integrating neuroscience with data-driven methodologies. This interdisciplinary field leverages machine learning algorithms, neuroimaging techniques (e.g., functional magnetic resonance imaging, fMRI), and large-scale biomedical datasets to develop objective diagnostic tools that complement traditional subjective clinical assessments. Recent advancements in neuroimaging and genomics have significantly improved the accuracy of psychiatric disorder classification, offering a viable alternative to conventional subjective evaluations. Building on these advancements, the present study introduces an innovative multimodal framework designed to accurately distinguish among SZ, BD, and healthy controls (HC).

Recent studies have demonstrated progressive improvements in differentiating psychiatric disorders through machine learning approaches (Tikka et al., 2020). Conventional machine learning methods, such as support vector machines (SVMs), random forests, and logistic regression, have shown moderate classification performance, typically achieving accuracies ranging from 70% to 85% (Chen et al., 2024; Emre et al., 2023). For instance, Tikka et al. reported an accuracy of 78.95% in discriminating SZ patients from healthy controls using an SVM classifier (Tikka et al., 2020). However, these traditional approaches exhibit significant limitations in multiclass classification tasks: for example, only 70.8% accuracy was reported when distinguishing among SZ, BD, and healthy controls (Chen et al., 2024). A systematic comparison of seven psychiatric disorders further demonstrated that classification accuracies across various algorithms ranged from 75% to 85% (Emre et al., 2023), highlighting the suboptimal performance of conventional methods in multi-disorder discrimination.

Beyond multiclass limitations, these traditional methods also face inherent shortcomings in capturing complex neurobiological patterns, primarily due to their reliance on manually engineered features. In contrast, deep learning approaches have demonstrated superior performance in psychiatric classification tasks (Shams and Jabbari, 2024; Du et al., 2020; Guo et al., 2024). In binary classification (e.g., patient vs. healthy control), exceptional accuracy of 99% has been achieved using convolutional neural network (CNN) models (Shams and Jabbari, 2024). For multiclass applications, accuracies of 85% have been reported in distinguishing SZ and autism spectrum disorder (ASD) (Du et al., 2020), while combined 3D/2D-CNN architectures have reached 89.85% accuracy for SZ identification (Guo et al., 2024). Although multiclass performance remains below binary classification benchmarks, these results represent significant improvements over traditional machine learning methods. Notably, studies involving limited sample sizes have highlighted the data dependency of deep learning models, reporting accuracies of 78.2% for BD and 71.0% for SZ (Chen et al., 2025)—this underscores the critical need for approaches that are robust to small-sample constraints, a common challenge in psychiatric neuroimaging research.

To address these aforementioned limitations, we propose an innovative computational framework that integrates several methodological advances. First, we introduce data augmentation for small-sample learning. Second, we developed specialized dual-path CNN architectures for gray matter volume (GMV) and fractional amplitude of low-frequency fluctuations (fALFF) feature extraction, specifically optimized to accommodate the unique characteristics of neuroimaging data. Finally, we implement evidence-theoretic multimodal fusion of classification probabilities using Dempster-Shafer (D-S) theory to quantify diagnostic certainty and enhance classification robustness. This comprehensive approach simultaneously addresses two critical challenges in psychiatric neuroimaging: achieving reliable classification with limited training data and maintaining clinical interpretability through neurobiologically meaningful feature selection.

The remainder of this paper is organized as follows. Section 2 presents our methodological framework, detailing the three key innovations: (1) the augmentation algorithm, (2) dual-path CNN architecture design for feature extraction, and (3) evidence-theoretic fusion strategy. Section 3 describes the experimental validation, including performance evaluation under five-fold cross-validation with limited sample sizes. Finally, Section 4 provides a comprehensive summary of the entire study, encapsulating the key findings, methodological innovations, and clinical implications of our work.

## 2 Methods

This study was conducted in strict compliance with the ethical principles outlined in the Declaration of Helsinki, which governs medical research involving human subjects, and all procedures were designed to protect the rights, safety, and well-being of the participants. The study protocol was reviewed and formally approved by the Ethics Committee of Hangzhou Seventh People's Hospital, ensuring that all research activities met the highest ethical standards. The study commenced in January 2022 and concluded in December 2024. Prior to their participation, all individuals were provided with a comprehensive and clear description of the study's purpose, procedures, potential risks, benefits, and the right to withdraw from the study at any time without any adverse consequences. After fully understanding the study details, all participants voluntarily signed a written informed consent form, which was properly filed and preserved for ethical and legal verification.

The dataset used in this study was carefully curated and included clinical and neuroimaging data from 31 patients with schizophrenia (SZ; 16 males/15 females; age: 45.13 ± 5.33 years), 48 patients with bipolar I disorder (BD-I; 27 males/21 females; age: 43.63 ± 9.73 years), and 38 healthy controls (HC; 20 males/18 females; age: 45.71 ± 9.63 years), with the control group matched as closely as possible to the patient groups in terms of age and educational level. Diagnoses of SZ and BD-I were established according to DSM-5 criteria and confirmed by structured clinical interviews. All participants were right-handed Han Chinese adults aged 18–65 years. Patients were clinically stable and receiving commonly used antipsychotic or mood-stabilizing medications within therapeutic dose ranges. Individuals with major neurological disorders, serious systemic diseases affecting cognitive function, substance abuse or dependence, recent MECT or TMS treatment, or contraindications to MRI were excluded. The study was approved by the institutional ethics committee, and all participants provided written informed consent. All participant information was de-identified to protect privacy; personal identifiers, including names, medical record numbers, and contact information, were removed or encrypted to prevent direct or indirect identification of individual participants.

Throughout the study, we adhered to the principles of confidentiality, autonomy, and beneficence. The collected data were only used for the research purposes stated in the study protocol and were not shared with any third parties without additional ethical approval. All experimental procedures involving the use of participant data were conducted in accordance with the approved protocol, and every effort was made to minimize any potential discomfort or risk to the participants. The ethical review committee conducted periodic oversight of the study to ensure ongoing compliance with ethical guidelines, and any modifications to the study protocol were submitted for re-approval prior to implementation.

All participants were scanned by a 3T MR system (GE HDx, Chicago, Illinois) with an eight-channel phased array head coil in the Hangzhou Seventh People's Hospital, Hangzhou, China. T1-weighted images were acquired by three-dimensional spoiled gradient echo sequence as follows: repetition time (TR) = 11.94ms, echo time (TE) = 5.044ms, flip angle = 15°, slice thickness =1mm without gap, number of slices =172, field of view (FOV) = 240 × 240 mm, matrix size = 256 × 256. In addition, R-fMRI data were acquired with a gradient recalled echo echo-planar imaging (GRE-EPI) sequence: repetition time (TR) = 2000ms, echo time (TE) = 25ms, flip angle = 90°, number of slices = 35, field of view (FOV) = 240 × 240 mm, slice thickness = 4 mm without gap, matrix size = 64 × 64, voxel size = 4 × 4 × 4 mm3, 240 volumes. All participants were asked to lie quietly awake in the scanner with eyes closed, and their heads were cozily positioned with cushions inside the coil during the MRI scan to minimize head motion.

The Statistical Parametric Mapping 8 (SPM8, http://www.fil.ion.ucl.ac.uk/spm/software/spm8/) and Data Processing & Analysis for Brain Imaging (DPABI, http://rfmri.org/dpabi) were applied to preprocess the fMRI data in MATLAB (http://www.mathworks.com/products/matlab/). The data preprocessing includes the following steps: (1) discarding of the first 10 volumes to achieve equilibrium and a steady-state; (2) slice timing correction; (3) realignment (head motion parameters were computed by estimating the translation in each direction and the angular rotation on each axis for each volume, we required the translational or rotational motion parameters less than 3 mm or 3°, The frame-wise displacement (FD), which indexes the volume-to-volume changes in head position was also calculated; (4) spatially normalized: individual structural images were firstly co-registered with the mean functional image; then the transformed structural images were segmented and normalized to the Montreal Neurological Institute (MNI) space using a high-level nonlinear warping algorithm which use the exponentiated Lie algebra (DARTEL) technique to acquire the diffeomorphic anatomical registration, Finally, each functional volume was spatially normalized to MNI space using the deformation parameters estimated during the above step and resampled into a 3-mm cubic voxel; (5) Nuisance covariates regression: six head motion parameters, cerebrospinal fluid signals, white matter signals, and global mean signals were regressed from the data as corrected values; (6)spatial smoothing with a Gaussian kernel of 6mm × 6mm × 6mm.

To overcome the difficulties in obtaining medical data samples and the low identification accuracy of conventional methods, we propose a multi-modal fusion recognition method, which effectively achieves the identification of SZ, BD patients and healthy controls. The algorithmic framework of this study is shown in Figure 1.

**Figure 1:**
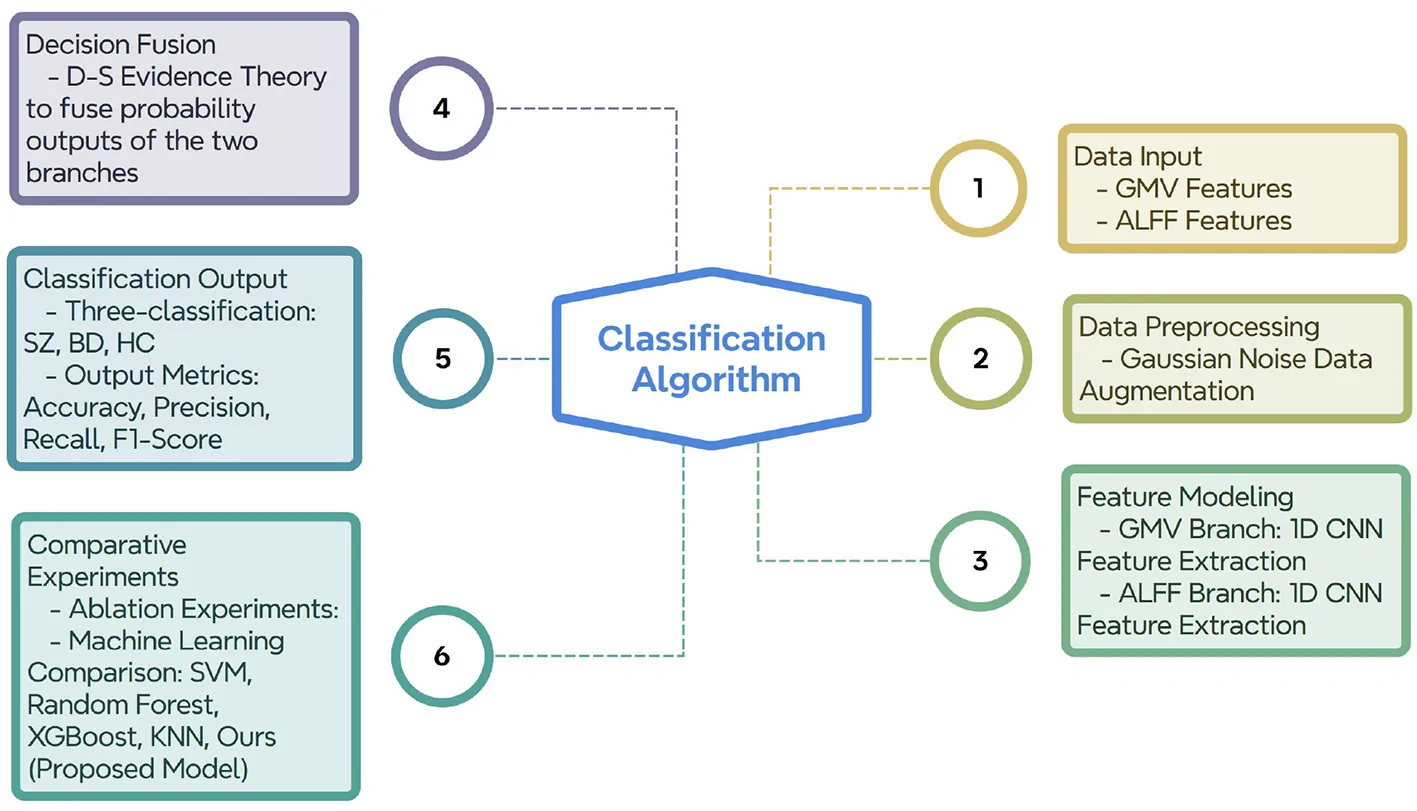
Framework diagram of the discriminative model based on data augmentation and data fusion.

This comprehensive framework addresses the critical challenge of limited sample sizes in psychiatric neuroimaging through three synergistic innovations: data augmentation, deep learning-based discriminative, and evidence-theoretic decision fusion. Each component is designed to enhance model robustness while maintaining neurobiological interpretability.

### 2.1 Data augmentation

To address the limitations of small sample sizes, we developed an data augmentation framework that maintains the essential structural and functional relationships of the brain (Huang et al., 2022). As illustrated in Figure 2, our augmentation framework operates on the original dataset, formally defined as $D^{\left(0\right)}=\left\{D_{SZ}^{\left(0\right)},D_{BD}^{\left(0\right)},D_{HC}^{\left(0\right)}\right\}$, where each diagnostic group *c*∈{*SZ, BD, HC*} comprises *n*_*c*_ samples denoted by $D_{c}^{\left(0\right)}={\left\{\left(X_{j}^{\left(1\right)},X_{j}^{\left(2\right)},y_{j}\right)\right\}}_{j=1}^{n_{c}}$. Here, $X_{j}^{\left(1\right)}=\left(x_{j1}^{\left(1\right)},x_{j2}^{\left(1\right)},\ldots ,x_{jp}^{\left(1\right)}\right)\in R^{p}$ and $X_{j}^{\left(2\right)}=\left(x_{j1}^{\left(2\right)},x_{j2}^{\left(2\right)},\ldots ,x_{jp}^{\left(2\right)}\right)\in R^{p}$ represent the gray matter volume (GMV) and fractional amplitude of low-frequency fluctuations (fALFF) feature vectors, respectively, corresponding to *p* brain regions. The class label *y*_*j*_∈{0, 1, 2} is assigned to schizophrenia (SZ, *y*_*j*_ = 0), bipolar disorder (BD, *y*_*j*_ = 1), and healthy control (HC, *y*_*j*_ = 2).

**Figure 2:**
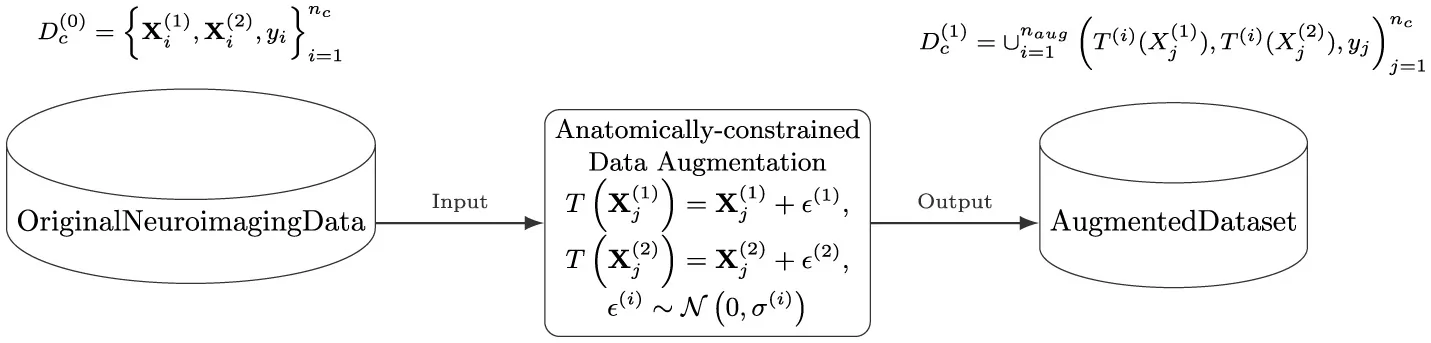
Neuroimaging data augmentation framework.

We formally define our augmentation transformation as a mapping $T:R^{p}\to R^{p}$. For an input sample from diagnostic group *c*, denoted by $D_{c}^{\left(0\right)}={\left\{\left(X_{j}^{\left(1\right)},X_{j}^{\left(2\right)},y_{j}\right)\right\}}_{j=1}^{n_{c}}$, the augmented sample set $D_{c}^{\left(1\right)}$ is generated as $D_{c}^{\left(1\right)}={\left\{\left(T\left(X_{j}^{\left(1\right)}\right),T\left(X_{j}^{\left(2\right)}\right),y_{j}\right)\right\}}_{j=1}^{n_{c}}$. Specifically, the transformation *T* implements additive Gaussian noise, which is formulated as follows (Equation 1):

$$\{\begin{array}{c} T\left(X_{j}^{\left(1\right)}\right)=X_{j}^{\left(1\right)}+\epsilon^{\left(1\right)}\\ T\left(X_{j}^{\left(2\right)}\right)=X_{j}^{\left(2\right)}+\epsilon^{\left(1\right)} \end{array}$$ (1)

where $\epsilon^{\left(i\right)}\sim N\left(0,\sigma^{\left(i\right)}\right)$ for *i*∈{1, 2}. Here, σ^(*i*)^ denotes the covariance matrix that governs the noise distribution for each feature type, i.e., GMV and fALFF.

The augmented datasets are formally constructed as $D^{\left(1\right)}=\left\{D_{SZ}^{\left(1\right)},D_{BD}^{\left(1\right)},D_{HC}^{\left(1\right)}\right\}$ through the systematic application of our proposed transformation framework. For each diagnostic group *c*∈{*SZ, BD, HC*}, the augmented dataset $D_{c}^{\left(1\right)}$ can be derived following the procedure detailed below (Equation 2).

$$\begin{array}{lll} D_{c}^{\left(1\right)} & = & \cup_{i=1}^{n_{aug}}{\left\{\left(T^{\left(i\right)}\left(X_{j}^{\left(1\right)}\right),T^{\left(i\right)}\left(X_{j}^{\left(2\right)}\right),y_{j}\right)\right\}}_{j=1}^{n_{c}} \end{array}$$

$$\begin{array}{ll} = & {\left\{\left(X_{j}^{\left(1*\right)},X_{j}^{\left(2*\right)},y_{j}\right)\right\}}_{j=1}^{n_{c}^{*}}, \end{array}$$ (2)

where *n*_*aug*_ represents the predetermined augmentation multiplicity factor, *T*^(*i*)^ denotes the *i*-th application of our constrained transformation. Total augmented sample per class is $n_{c}^{*}=n_{c}\times n_{aug}$.

To ensure clarity and reproducibility, we provide detailed implementation settings for the Gaussian augmentation used in this study. *n*_*aug*_ augmented samples were generated for each original training sample, effectively doubling the size of the training set after augmentation. Only one independent Gaussian noise realization was applied to each sample during augmentation, rather than multiple repeated perturbations.

Prior to CNN training, all feature vectors were standardized using Z-score normalization. Specifically, for each modality (GMV and ALFF) independently, we subtracted the mean and divided by the standard deviation, resulting in features with zero mean and unit variance. This step eliminates scale differences between GMV and ALFF features that could otherwise affect multimodal fusion performance.

It should be noted that data augmentation was applied to the training data only, with details provided in Section 3.

### 2.2 1D-CNN model classification

Recently, convolutional Neural Networks (CNNs) have emerged as powerful discriminative models in deep learning, exhibiting exceptional performance across various domains owing to their inherent ability to extract features and learn efficiently automatically. In the medical field, CNNs have been widely and successfully applied to disease classification tasks (Latreche et al., 2024; Pan et al., 2024). To fully exploit the complementary information of GMV and fALFF features, we implement two parallel 1D-CNN architectures, each dedicated to processing GMV and fALFF features, respectively.

For the GMV feature vectors **X**^(1*)^ (i.e., GMV data after data augmentation), defined as $X^{\left(1*\right)}=\left(x_{1}^{\left(1*\right)},x_{2}^{\left(1*\right)},\ldots ,x_{L}^{\left(1*\right)}\right)\in R^{n^{*}\times 1\times L}$, where n^*^ denotes the number of samples after data augmentation, *L* denotes the length of GMV feature vectors, the forward propagation process through the 1D-CNN is mathematically formulated as follows.

The first convolutional layer employs 16 filters with a kernel size of 3, which is specifically chosen to capture local gyral patterns in gray matter distribution (Madububambachu et al., 2024). The convolution operation is expressed as Equation 3:

$$\begin{array}{l} H_{gmv}^{\left(1\right)}=\text{ReLU}\left(W_{gmv}^{\left(1\right)}*X^{\left(1*\right)}+B_{gmv}^{\left(1\right)}\right), \end{array}$$ (3)

where $W_{gmv}^{\left(1\right)}\in R^{16\times 3}$ denotes the convolution filters, $B_{gmv}^{\left(1\right)}\in R^{16}$ are bias terms, and the output $H_{gmv}^{\left(1\right)}\in R^{n^{*}\times 1\times L}$.

Subsequently, a max-pooling layer with a pooling size and stride of 2 is applied to reduce dimensionality while preserving key features. The output of this pooling layer $H^{\left(2\right)}\in R^{n^{*}\times 16\times \left\lfloor \frac{L}{2}\right\rfloor }$ is given by Equation 4:

$$\begin{array}{l} h_{gmv}^{\left(2\right)}\left[c,j\right]=max\left(h_{gmv}^{\left(1\right)}\left[c,2j-1\right],h_{gmv}^{\left(1\right)}\left[c,2j\right]\right). \end{array}$$ (4)

The second convolutional block further integrates deep features using 32 convolutional filters, maintaining a kernel size of 3 to retain the same receptive field while increasing channel depth to capture more complex, higher-order patterns across brain regions. This operation is defined as Equation 5:

$$\begin{array}{l} H_{gmv}^{\left(3\right)}=\text{ReLU}\left(W_{gmv}^{\left(2\right)}*H_{gmv}^{\left(2\right)}+B_{gmv}^{\left(2\right)}\right). \end{array}$$ (5)

where $W_{gmv}^{\left(2\right)}\in R^{32\times 16\times 3}$ are the convolution filters, $B_{gmv}^{\left(2\right)}\in R^{32}$ are bias terms, and the output $H_{gmv}^{\left(3\right)}\in R^{n^{*}\times 32\times \left\lfloor \frac{L}{2}\right\rfloor }$.

A second max-pooling layer is applied for critical dimensionality reduction, with its output $H^{\left(4\right)}\in R^{n^{*}\times 32\times \left\lfloor \frac{L}{4}\right\rfloor }$ calculated as Equation 6:

$$\begin{array}{l} h_{gmv}^{\left(4\right)}\left[c,k\right]=max\left(h_{gmv}^{\left(3\right)}\left[c,2k-1\right],h_{gmv}^{\left(3\right)}\left[c,2k\right]\right). \end{array}$$ (6)

Following the pooling layer, the spatio-temporal features are flattened into a vector representation, which is then progressively transformed through three successive fully connected layers. The flattened feature vector is Equation 7:

$$\begin{array}{l} f=\text{Flatten}\left(H^{\left(4\right)}\right)\in R^{n^{*}\times \left(32\times \left\lfloor \frac{L}{4}\right\rfloor \right)}. \end{array}$$ (7)

The first fully connected layer performs feature transformation, with its output computed as Equation 8:

$$\begin{array}{lll} {Fc}_{gmv}^{\left(1\right)} & = & \text{ReLU}\left(W_{fc}^{\left(1\right)}f+B_{fc}^{\left(1\right)}\right)\in R^{n^{*}\times 128},W_{fc}^{\left(1\right)}\in R^{128\times \left(32\times \left\lfloor \frac{L}{4}\right\rfloor \right)}, \end{array}$$

$$\begin{array}{lll} B_{fc}^{\left(1\right)} & \in & R^{128}. \end{array}$$ (8)

A dimension reduction layer further processes the features, yielding (Equation 9):

$$\begin{array}{lll} {Fc}_{gmv}^{\left(2\right)} & = & \text{ReLU}\left(W_{fc}^{\left(2\right)}{Fc}_{gmv}^{\left(1\right)}+B_{fc}^{\left(2\right)}\right)\in R^{n^{*}\times 64},W_{fc}^{\left(2\right)}\in R^{64\times 128}, \end{array}$$

$$\begin{array}{lll} B_{fc}^{\left(2\right)} & \in & R^{64}. \end{array}$$ (9)

Finally, the output projection layer produces the logits for three classes (SZ, BD, HC) as seen in the Equation 10:

$$\begin{array}{l} Z^{\left(1\right)}=W_{fc}^{\left(3\right)}{Fc}_{gmv}^{\left(2\right)}+B_{fc}^{\left(3\right)}\in R^{n^{*}\times 3},W_{fc}^{\left(3\right)}\in R^{3\times 64},B_{fc}^{\left(3\right)}\in R^{3}. \end{array}$$ (10)

The same 1D-CNN architecture and training procedure are adopted for processing fALFF data, where **X**^(2*)^ denotes the fALFF feature vectors after data augmentation, yielding the class probabilities $P\left(c|X_{j}^{\left(2*\right)}\right)$.

A softmax activation function is applied to the logits to obtain the class probability distribution. Let $Z^{\left(1\right)}=\left(Z_{1}^{\left(1\right)},Z_{2}^{\left(1\right)},\ldots ,Z_{n^{*}}^{\left(1\right)}\right)$ denote the output of the final fully connected layer before softmax, where $Z_{j}^{\left(1\right)}=\left(z_{j,SZ}^{\left(1\right)},z_{j,BD}^{\left(1\right)},z_{j,HC}^{\left(1\right)}\right)$. The probability that the *j*-th sample belongs to class *c*∈{*SZ, BD, HC*} is Equation 11.

$$\begin{array}{l} P\left(c|X_{j}^{\left(1*\right)}\right)=\frac{e^{z_{j,c}^{\left(1\right)}}}{\underset{c}{\sum }e^{z_{j,c}^{\left(1\right)}}}. \end{array}$$ (11)

The same architecture and training procedure are applied to construct the CNN model for fALFF data. Similarly, we can also obtain the probabilities of each category under the discrimination of fALFF $P\left(c|X_{j}^{\left(2*\right)}\right)$.

During traning, the model parameters are optimized by minimizing a cross-entropy loss function (Equation 12).

$$\begin{array}{l} L=-\overset{n^{*}}{\underset{i=1}{\sum }}\overset{2}{\underset{k=0}{\sum }}I\left(y_{j}=k\right)\log\left(P\left(y_{j}=k|X_{j}^{\left(i*\right)}\right)\right). \end{array}$$ (12)

where *I*(·) denotes the indicator function (i.e., *I*(*y*_*j*_ = *k*) = 1 if *y*_*j*_ = *k*, otherwise *I*(*y*_*j*_ = *k*) = 0), *y*_*j*_ denotes the true label of the *j*-th sample, and the Adam optimizer is employed to update the weights and biases of the dual-branch CNN models.

The proposed 1D-CNN model consists of two 1D convolutional layers, two max-pooling layers, and three fully connected layers. The convolutional layers employ 16 and 32 filters, respectively, each with a kernel size of 3. The model is trained using the Adam optimizer with a learning rate of 0.001, a batch size of 32, and a maximum of 100 epochs. The loss function is categorical cross-entropy for the three-class classification task. No dropout or weight decay is used in the current implementation.

### 2.3 D-S fusion

The Dempster-Shafer (D-S) evidence theory provides a principled mathematical framework for combining probabilistic evidence from multiple sources from multiple neuroimaging modalities (GMV and fALFF) while explicitly quantifying and managing uncertainty (Zhu et al., 2022; Wang et al., 2024). For our multimodal classification problem, we implement the fusion process to combine predictions from two distinct feature domains.

Given probability outputs from two 1D-CNN models, let $P\left(c|X_{j}^{\left(1*\right)}\right)$ and $P\left(c|X_{j}^{\left(2*\right)}\right)$ denote the class probabilities from GMV features and fALFF features, respectively.

The D-S combination rule computes the fused probability mass (Equation 13).

$$\begin{array}{l} P\left(c|X_{j}^{\left(1*\right)}\right)⊕P\left(c|X_{j}^{\left(2*\right)}\right)=\frac{P\left(c|X_{j}^{\left(1*\right)}\right)•P\left(c|X_{j}^{\left(2*\right)}\right)}{P\left(c|X_{j}^{\left(1*\right)}\right)\cdot P\left(c|X_{j}^{\left(2*\right)}\right)} \end{array}$$ (13)

where ° denotes the element-wise product of the two probability mass functions, and · in the denominator represents the dot product. The denominator serves as a normalization factor that explicitly accounts for the total agreement between the two evidence sources. Specifically, let $A=P\left(c|X_{j}^{\left(1*\right)}\right)\cdot P\left(c|X_{j}^{\left(2*\right)}\right)$ denote the sum of products for matching class assignments between the GMV and ALFF modalities. The normalization factor 1/*A* ensures that the fused probability masses sum to unity. When *A* = 1 (i.e., perfect agreement with identical distributions), the combination reduces to a simple normalized product; when *A* = 0 (complete disagreement, no overlapping support), the combination is undefined, indicating that the two evidence sources are entirely contradictory.

We demonstrate the individual classification process through a concrete example of D-S evidence fusion. Let the probability outputs of two CNN models be *P*(*c*|**X**^(1*)^) = (0.7, 0.2, 0.1) and *P*(*c*|**X**^(2*)^) = (0.4, 0.5, 0.1).

Firstly, we calculate the element-wise product of the two probability outputs (Equation 14):

$$\begin{array}{l} P\left(c|X_{j}^{\left(1*\right)}\right)•P\left(c|X_{j}^{\left(2*\right)}\right)=\left(0.28,0.10,0.01\right) \end{array}$$ (14)

Then, we calculate the dot product (total agreement mass) (Equation 15):

$$\begin{array}{l} A=P\left(c|X_{j}^{\left(1*\right)}\right)\cdot P\left(c|X_{j}^{\left(2*\right)}\right)=0.28+0.10+0.01=0.39 \end{array}$$ (15)

Finally, the fused probability masses are obtained as follows (Equation 16):

$$\begin{array}{lll} P\left(c|X_{j}^{\left(1*\right)}\right)⊕P\left(c|X_{j}^{\left(2*\right)}\right) & = & \frac{\left(0.28,0.10,0.01\right)}{0.39} \end{array}$$

$$\begin{array}{ll} = & \left(0.7179,0.2564,0.0256\right) \end{array}$$ (16)

Initial unimodal classification results exhibited diagnostic disagreement, with structural GMV features strongly supporting a SZ classification while functional fALFF indicators suggested BD. The D-S evidence fusion framework successfully harmonized these conflicting predictions through its principled approach to combining probabilistic evidence.

The fusion process generated a unified probabilistic assessment, yielding a posterior probability distribution that clearly favored SZ with 71.8% confidence, compared to 25.6% for BD and 2.6% for HC. This outcome demonstrates the framework's capacity to quantitatively reconcile divergent diagnostic evidence while preserving the relative strength of each modality's predictive contribution.

## 3 Experiments and results

To rigorously evaluate the performance of our proposed algorithm, we conducted comprehensive experiments using a carefully curated dataset comprising 31 SZ patients, 48 BD patients, and 38 HC. The brain was parcellated into 246 ROIs using a predefined atlas. GMV and ALFF features were extracted for each ROI, yielding feature vectors of dimension *p* = 246 for both modalities. For multi-modal fusion, the GMV and ALFF features were combined at the ROI level, preserving the same dimensionality. A five-fold cross-validation experiment was conducted at the subject level, ensuring that all data from the same subject were assigned to a single fold. Data augmentation was applied only to the training set after splitting, and the model was evaluated on the original, non-augmented test set, thereby preventing data leakage. Data augmentation was performed within each fold of the five-fold cross-validation, applied exclusively to the training subset after each train/test split, while the test portion remained unmodified. This fold-wise scheme ensures that augmented samples never contaminate the test set, thereby preventing information leakage and enabling an unbiased evaluation of generalization performance. The detailed results of the five-fold cross-validation for the proposed model are presented as follows, where all evaluation metrics are reported as the mean value plus or minus the standard deviation, reflecting the stability and reliability of the model.

All experiments were conducted under the following hardware and software configurations, Operating System: Windows 10 Professional; Programming Language: Python 3.9; Deep Learning Framework: PyTorch 1.12.1; Machine Learning Libraries: scikit-learn 1.0.2, XGBoost 1.5.2; Parallel Computing: CUDA 11.3 and cuDNN 8.2; Graphics Processing Unit (GPU): NVIDIA RTX 3060.

### 3.1 Analysis of model performance

First, the calculation formulas of the four key evaluation metrics (Accuracy, Precision, Recall, and F1-Score) are defined as follows. Here, *TP* (True Positive) denotes the number of correctly predicted positive samples, *TN* (True Negative) denotes the number of correctly predicted negative samples, *FP* (False Positive) denotes the number of incorrectly predicted positive samples, and *FN* (False Negative) denotes the number of incorrectly predicted negative samples. The specific formulas are (Equations 17–20):

$$\begin{array}{lll} \text{Accuracy} & = & \frac{TP+TN}{TP+TN+FP+FN} \end{array}$$ (17)

$$\begin{array}{lll} \text{Precision} & = & \frac{TP}{TP+FP} \end{array}$$ (18)

$$\begin{array}{lll} \text{Recall} & = & \frac{TP}{TP+FN} \end{array}$$ (19)

$$\begin{array}{lll} \text{F1-Score} & = & 2\times \frac{\text{Precision}\times \text{Recall}}{\text{Precision}+\text{Recall}} \end{array}$$ (20)

For multiclass evaluation, all metrics were computed using micro-averaging, where the confusion matrices of all classes are pooled before calculating Precision, Recall, and F1-Score. Notably, under micro-averaging, Precision, Recall, and F1-Score are numerically equal to overall Accuracy.

To obtain the above cross-validation results, we first performed data augmentation on the original data, followed by CNN probability calculation, and finally adopted Dempster-Shafer (DS) fusion for result integration.

To explore the influence of Gaussian noise intensity and augmentation quantity on model performance, we conduct ablation experiments on two key hyperparameters: noise standard deviation σ and augmentation multiplicity. We select three noise levels σ∈{0.05, 0.1, 0.2} and three augmentation multiplicities {1, 5, 10}, where the multiplicity of 1 represents the original training set without data augmentation, which is regarded as the baseline.

All experiments adopt 5-fold cross-validation with a fixed random seed of 42 for full reproducibility. The network structure, Adam optimizer (learning rate = 0.001), batch size (32) and maximum training epoch (100) keep consistent across all groups.

As observed from the above Figure 3, the highest F1-score is obtained when the noise standard deviation σ = 0.2 and the augmentation multiplicity is set to 10. Thus, we adopt this set of hyperparameters for all subsequent experiments.

**Figure 3:**
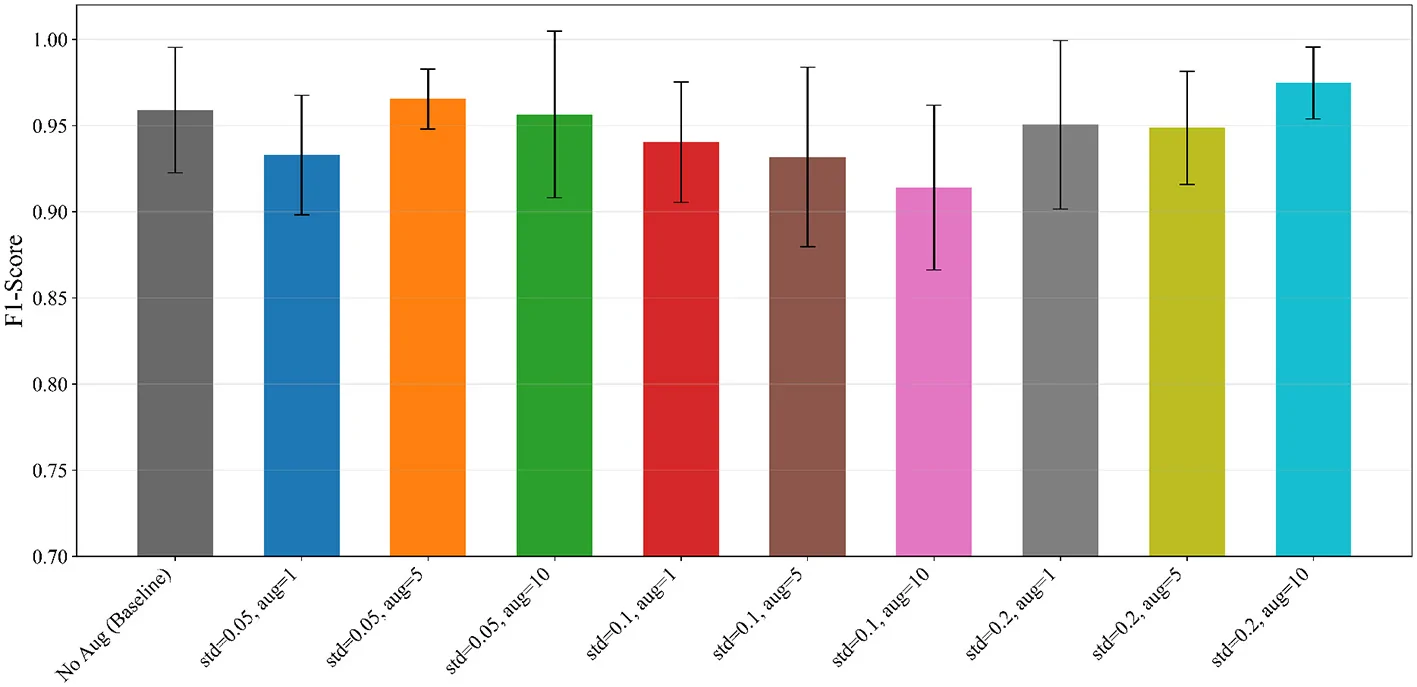
F1-score under different Gaussian noise standard deviations and augmentation multiplicities.

After the completion of the five-fold cross-validation experiment, the boxplots visualizing the distribution of various evaluation metrics are shown in Figure 4.

**Figure 4:**
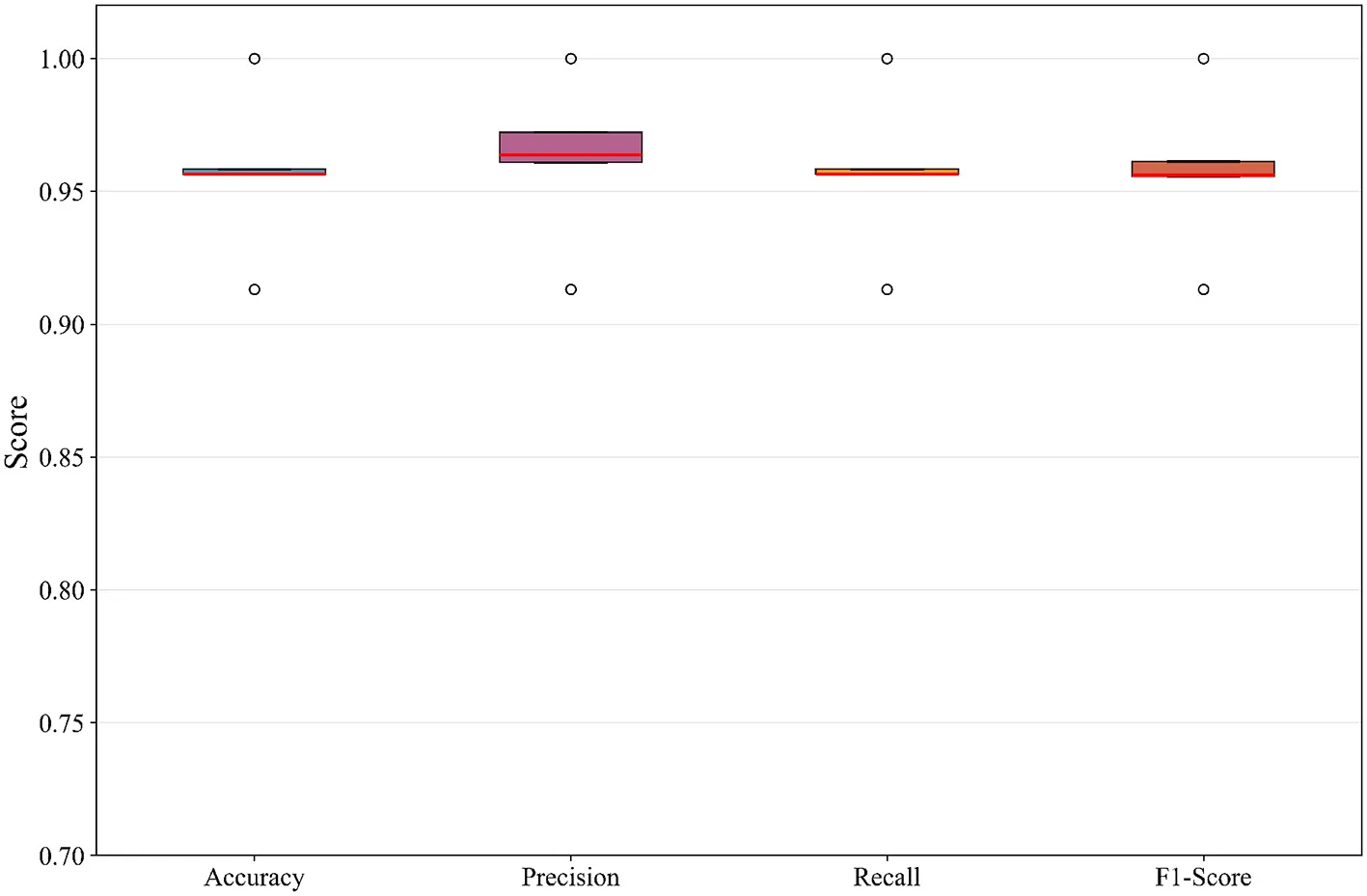
5-fold cross validation metrics distribution.

Specifically, the proposed full model (Ours (Full Model)) achieved excellent performance across all four key evaluation metrics. For the Accuracy metric, which measures the overall classification correctness of the model, the result was 0.9656±0.0324, indicating that the model correctly classified 96.56% of the samples on average, with a standard deviation of 0.0324, demonstrating good stability in classification accuracy. In terms of Precision, which reflects the proportion of true positive samples among all samples predicted as positive, the model obtained a result of 0.9698±0.0319, showing that the model has high accuracy in identifying positive samples and a low false positive rate. For the Recall metric, which represents the proportion of true positive samples among all actual positive samples, the result was 0.9656±0.0324. Finally, the F1-Score, which is the harmonic mean of Precision and Recall and comprehensively reflects the balance between the two metrics, was 0.9661±0.0324. This high F1-Score further confirms that the proposed full model has excellent comprehensive classification performance, with good balance between precision and recall, and strong generalization ability to unseen data.

We further plotted the confusion matrix and receiver operating characteristic (ROC) curves for quantitative evaluation in Figures 5, 6.

**Figure 5:**
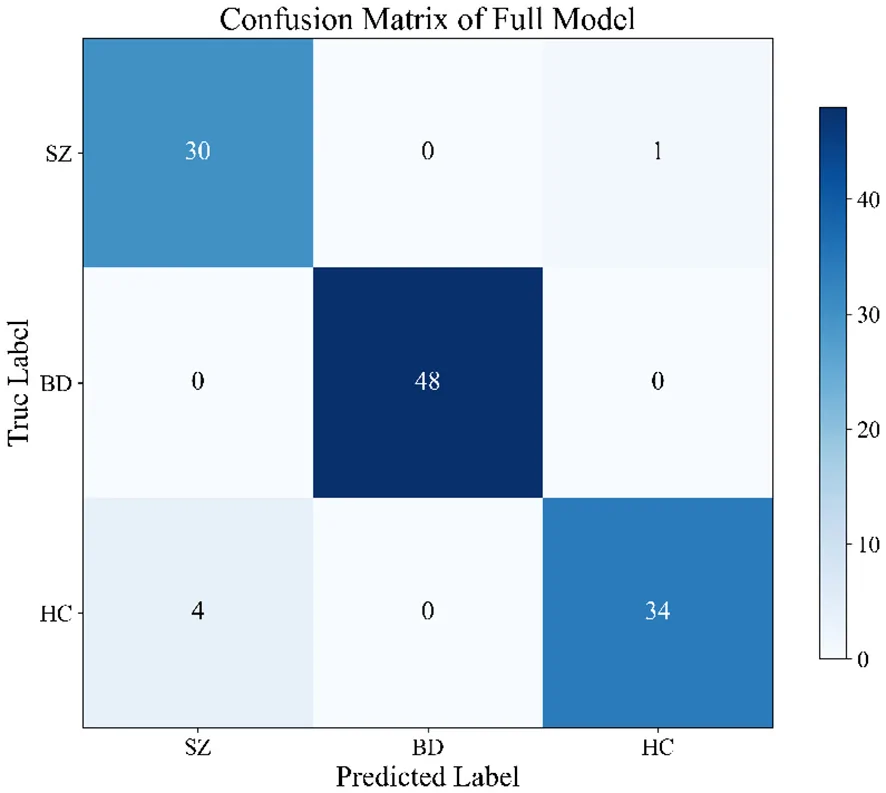
5-Fold Cross validation metrics distribution.

**Figure 6:**
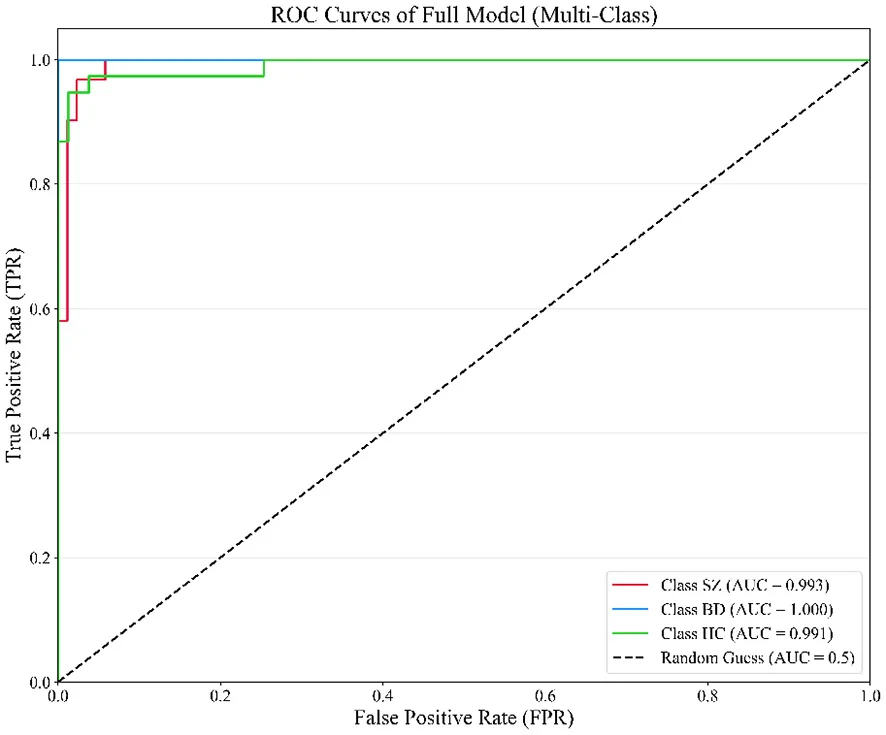
ROC curves of full model (multi-class).

The confusion matrix reveals that the model achieves perfect classification for BD (48/48), indicating that bipolar disorder has a distinct multimodal neuroimaging signature that is well-separated from both SZ and HC. The primary misclassifications occur between SZ and HC (1 SZ misclassified as HC, 4 HC as SZ), suggesting partial overlap in GMV and ALFF features between schizophrenia patients and healthy controls. This is likely due to the clinical heterogeneity of SZ–some patients may exhibit normalized or less severe brain alterations, making them appear similar to HC. The absence of any confusion between BD and the other two classes further supports the strong discriminability of BD in this dataset. Notably, no misclassification involves BD, confirming its distinct feature space.

The results demonstrate that all subjects with BD were correctly identified. Nevertheless, four HC subjects were misclassified as SZ, and one SZ subject was misclassified as HC. The area under the curve (AUC) values were 0.993 for SZ, 1.000 for BD, and 0.991 for HC, respectively.

The sensitivity, specificity, precision, and F1-score of each category and their 95% bootstrap confidence intervals are presented in the Table 1.

**Table 1:** Per-class performance metrics of the full model (mean±std with 95% bootstrap confidence intervals).

Metric	SZ	BD	HC
Sensitivity	0.9750 ± 0.0500 [0.9250, 1.0000]	1.0000 ± 0.0000 [1.0000, 1.0000]	0.9211 ± 0.0751 [0.8578, 0.9833]
Specificity	0.9665 ± 0.0282 [0.9420, 0.9909]	1.0000 ± 0.0000 [1.0000, 1.0000]	0.9818 ± 0.0364 [0.9455, 1.0000]
Precision	0.8750 ± 0.1236 [0.7667, 0.9750]	1.0000 ± 0.0000 [1.0000, 1.0000]	0.9833 ± 0.0333 [0.9500, 1.0000]
F1-Score	0.9168 ± 0.0765 [0.8516, 0.9818]	1.0000 ± 0.0000 [1.0000, 1.0000]	0.9493 ± 0.0445 [0.9105, 0.9882]

Table 1 reports the per-class performance of the proposed model in terms of sensitivity, specificity, precision, and F1-Score, together with their 95% bootstrap confidence intervals (2,000 iterations, percentile method). The model achieved perfect classification for BD (all four metrics equal to 1.0000), confirming the distinct multimodal neuroimaging signature of bipolar disorder. For SZ, a high sensitivity of 0.9750 was obtained, while its precision was comparatively lower (0.8750), indicating that a small number of HC subjects were misclassified as SZ. For HC, the sensitivity and precision reached 0.9211 and 0.9833, respectively. These findings corroborate the confusion matrix analysis: misclassifications occurred mainly between SZ and HC, whereas BD was separated with perfect accuracy.

### 3.2 Ablation study

Furthermore, we also conducted an ablation study, the results of which are shown in the Table 2.

**Table 2:** Ablation study results.

Method	Accuracy	Precision	Recall	F1-Score
Only GMV	0.8551 ± 0.0420	0.8746± 0.0437	0.8551± 0.0420	0.8549 ± 0.0365
Only ALFF	0.9315 ± 0.0429	0.9523 ± 0.02923	0.9315 ± 0.0429	0.9344 ± 0.0392
GMV+ALFF+DS	0.9482 ± 0.0427	0.9545 ± 0.0414	0.9482 ± 0.0427	0.9488 ± 0.0424
Full Model	0.9656 ± 0.0324	0.9698 ± 0.0319	0.9656 ± 0.0324	0.9661 ± 0.0324

To systematically verify the individual and synergistic effects of gray matter volume (GMV) features, amplitude of low-frequency fluctuation (ALFF) features, D-S evidence fusion strategy, and Gaussian data augmentation strategy, four comparative schemes were designed for ablation experiments. Four classic classification metrics, including Accuracy, Precision, Recall, and F1-Score, were adopted for comprehensive evaluation. All results are presented as “mean ± standard deviation” based on 5-fold cross-validation to ensure the reliability and reproducibility of the experimental results. Overall, with the gradual addition of each effective module, the classification performance and prediction stability of the model showed a continuous improvement trend. The finally constructed Full Model achieved the optimal performance in all evaluation metrics, fully verifying the effectiveness of the proposed method.

**Comparative analysis of single-modal feature performance**

Single-modal experiments were conducted to explore the impact of individual features on model classification performance and clarify the differences in discriminative ability between different modal features:

- Using only GMV features (Only GMV): The model achieved the lowest performance in all metrics, with an Accuracy of 0.8551±0.0420 and an F1-Score of 0.8549±0.0365. This result indicates that the single GMV feature can only provide limited discriminative information, which is difficult to fully capture the essential differences among SZ, BD, and HC samples, and thus cannot meet the requirements of high-precision classification tasks.
- Using only ALFF features (Only ALFF): The model performance was significantly improved, with an Accuracy of 0.9315±0.0429, representing an absolute increase of 6.80% compared with the model using only GMV features. Meanwhile, Precision (0.9523±0.02923), Recall (0.9315±0.0429), and F1-Score (0.9344±0.0392) were also optimized synchronously with consistent improvement ranges.
- In this three-classification task, ALFF features have significantly stronger discriminative ability than GMV features, which can more effectively characterize the feature differences of the three types of samples and serve as a more advantageous single-modal input feature.

**Performance analysis of the full model**

On the basis of multi-modal fusion, the Gaussian data augmentation strategy was added to construct the core Full Model of this paper, and its performance achieved a qualitative leap.

Neither GMV nor ALFF single feature can achieve the optimal classification effect. Among them, the discriminative ability of ALFF features is significantly better than that of GMV features, providing a high-quality feature foundation for subsequent multi-modal fusion. It is worth noting that although the mean accuracy of the ALFF-only model and the proposed GMV+ALFF+DS full model are comparable (or identical), the full model achieves a smaller variance and is more stable across cross-validation folds. Multi-modal fusion is the key to improving model stability: The multi-modal feature fusion based on D-S evidence theory can effectively integrate the complementary information of GMV and ALFF, significantly reduce the model prediction fluctuation, and enhance the stability and robustness of the model. The data augmentation strategy has a significant effect: Gaussian data augmentation can effectively alleviate overfitting, fully tap the potential of data features, and work synergistically with multi-modal fusion to achieve a significant improvement in model performance. The Full Model has the optimal performance: The Full Model constructed in this paper, which integrates GMV+ALFF multi-modal features, D-S evidence fusion, and Gaussian data augmentation, achieved the best results in all four evaluation metrics. It fully verifies the effectiveness, superiority, and practicality of the proposed method, and can provide reliable technical support for the auxiliary diagnosis of mental diseases.

### 3.3 Comparison with machine learning models

To ensure a fair comparison, we systematically compared our proposed algorithm with well-established machine learning classifiers, namely K-Nearest Neighbors (KNN), Extreme Gradient Boosting (XGBoost), Random Forest, and SVM. All baseline models were evaluated under identical conditions, including the same Gaussian data augmentation strategy applied to the training data within the cross-validation framework. The detailed results are presented in Table 3.

**Table 3:** Performance comparison with other machine learning models.

Method	Accuracy	Precision	Recall	F1-Score
Full Model	0.9656 ± 0.0324	0.9698 ± 0.0319	0.9656 ± 0.0324	0.9661 ± 0.0324
SVM	0.8975 ± 0.0577	0.9257 ± 0.0451	0.8975 ± 0.0577	0.9012 ± 0.0534
RandomForest	0.8460 ± 0.0652	0.8787 ± 0.0681	0.8460 ± 0.0652	0.8495 ± 0.0643
XGBoost	0.8721 ± 0.0720	0.8927 ± 0.0678	0.8721 ± 0.0720	0.8756 ± 0.0700
KNN	0.8536 ± 0.0814	0.8970 ± 0.0694	0.8536 ± 0.0814	0.8499 ± 0.0878

To further validate the effectiveness and superiority of the proposed Full Model, a comparative experiment was conducted against four mainstream traditional machine learning algorithms: Support Vector Machine (SVM), Random Forest (RF), eXtreme Gradient Boosting (XGBoost), and K-Nearest Neighbor (KNN). All experimental results were obtained via 5-fold cross-validation and are presented as mean ± standard deviation (mean ± SD) of four core classification metrics: Accuracy, Precision, Recall, and F1-Score, ensuring the reliability and reproducibility of the results.

As shown in the performance compare table and graph (Table 2 and Figure 7), our Full Model outperforms all comparative traditional machine learning models across all evaluation metrics. Specifically, the Full Model achieves an Accuracy of 0.9656±0.0324, a Precision of 0.9698±0.0319, a Recall of 0.9656±0.0324, and an F1-Score of 0.9661±0.0324. Among the traditional methods, SVM performs the best, with an Accuracy of 0.8975±0.0577, a Precision of 0.9257±0.0451, and an F1-Score of 0.9012±0.0534. XGBoost and KNN exhibit comparable moderate performance, with Accuracies of 0.8721±0.0720 and 0.8536±0.0814, respectively. In contrast, RF demonstrates the poorest performance, with an Accuracy of only 0.8460±0.0652 and an F1-Score as low as 0.8495±0.0643, accompanied by large standard deviations indicating poor stability.

**Figure 7:**
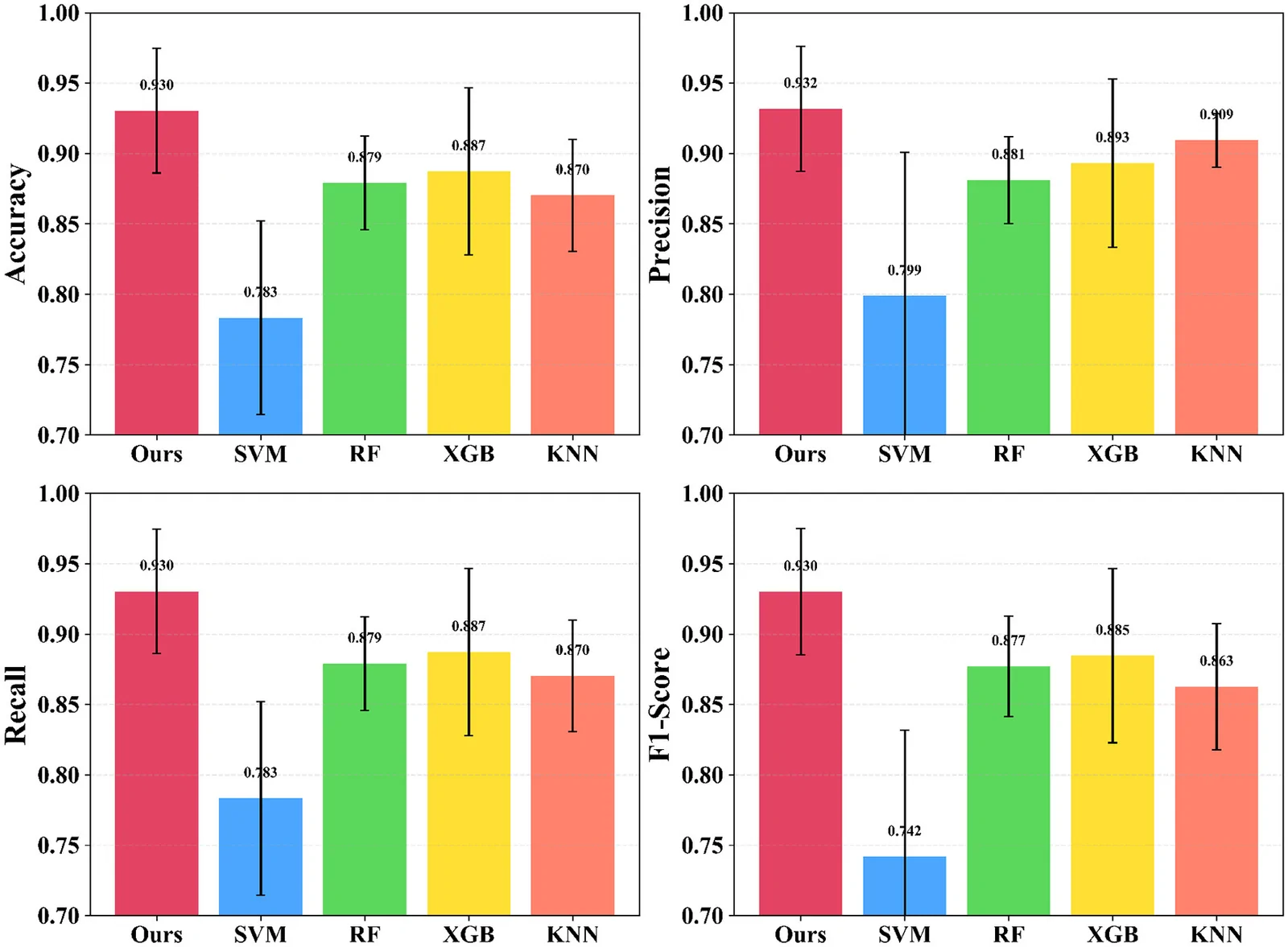
Comparison with other machine learning models.

The superior performance of the Full Model is primarily attributed to its integrated design: the fusion of GMV and ALFF multi-modal features via Dempster-Shafer (D-S) evidence theory effectively integrates complementary information from different brain imaging modalities, enhancing feature discriminability; the introduction of Gaussian data augmentation alleviates overfitting and improves generalization ability by expanding the training data distribution. In contrast, traditional machine learning models suffer from inherent limitations–SVM struggles to capture non-linear relationships in high-dimensional data, RF and KNN rely on shallow feature learning and manual feature engineering, and XGBoost, despite its regularization advantages, remains a shallow model unable to fully exploit deep multi-modal feature correlations.

In conclusion, the proposed Full Model exhibits significantly stronger feature representation and classification robustness than conventional machine learning methods, fully verifying its effectiveness and superiority in the three-category task of psychiatric disorder auxiliary diagnosis.

## 4 Conclusion

This study presents an innovative computational framework for psychiatric disorder classification that synergistically integrates four key methodological components: (1) advanced Gaussian data augmentation techniques to overcome sample size limitations and alleviate overfitting, (2) convolutional neural network (CNN) architectures for automated, end-to-end feature extraction from neuroimaging data, (3) Dempster-Shafer (D-S) evidence theory for robust decision fusion of multi-modal features, and (4) integration of structural (gray matter volume, GMV) and functional (amplitude of low-frequency fluctuation, ALFF) MRI biomarkers. The framework was specifically designed and validated for the challenging task of differentiating among schizophrenia (SZ), bipolar disorder (BD), and healthy control (HC) in small-sample neuroimaging datasets, with experimental results demonstrating its superior performance.

Our approach addresses two critical challenges in computational psychiatry: the scarcity of labeled medical imaging data that typically hinders deep learning applications, and the need for robust multi-modal integration of complementary neurobiological features. To tackle these challenges, the proposed solution combines structural (GMV) and functional (ALFF) MRI biomarkers through a novel fusion paradigm based on D-S evidence theory, demonstrating significant improvements over unimodal approaches. Specifically, ablation experiments confirmed that the fusion of GMV and ALFF features outperformed single-modal models, while the integration of data augmentation further enhanced performance.

Future work will focus on enhancing model interpretability by correlating machine learning outputs with clinical-pathological characteristics, aiming to elucidate neurobiological distinctions between SZ and BD and establish objective neuroimaging biomarkers for treatment evaluation. Additionally, efforts will be made to validate the framework on larger, multi-center datasets to improve its generalizability and clinical applicability.

## Data Availability

The data analyzed in this study is subject to the following licenses/restrictions: Any request for the corresponding data via email must be approved by the ethics committee. Requests to access these datasets should be directed to zwjnijiou@163.com.
